# *Suppressor of cytokine signaling 1* gene mutation status as a prognostic biomarker in classical Hodgkin lymphoma

**DOI:** 10.18632/oncotarget.4829

**Published:** 2015-08-20

**Authors:** Jochen K. Lennerz, Karl Hoffmann, Anna-Maria Bubolz, Davor Lessel, Claudia Welke, Nele Rüther, Andreas Viardot, Peter Möller

**Affiliations:** ^1^ Ulm University, Institute of Pathology, Ulm, Germany; ^2^ Massachusetts General Hospital/Harvard Medical School, Department of Pathology, Center for Integrated Diagnostics, Boston, MA, USA; ^3^ Department of Dermatology and Venerology, University of Freiburg Medical Center, Freiburg, Germany; ^4^ Ulm University, Institute of Human Genetics, Ulm, Germany; ^5^ University Medical Center Hamburg-Eppendorf, Institute of Human Genetics, Hamburg, Germany; ^6^ Comprehensive Cancer Center, Ulm University, Ulm, Germany; ^7^ Ulm University, Department of Internal Medicine III, Ulm, Germany

**Keywords:** SOCS1, Hodgkin lymphoma, cHL, prognostic biomarker

## Abstract

*Suppressor of cytokine signaling 1* (*SOCS1*) mutations are among the most frequent somatic mutations in classical Hodgkin lymphoma (cHL), yet their prognostic relevance in cHL is unexplored. Here, we performed laser-capture microdissection of Hodgkin/Reed-Sternberg (HRS) cells from tumor samples in a cohort of 105 cHL patients. Full-length *SOCS1* gene sequencing showed mutations in 61% of all cases (*n* = 64/105). Affected DNA-motifs and mutation pattern suggest that many of these *SOCS1* mutations are the result of aberrant somatic hypermutation and we confirmed expression of mutant alleles at the RNA level. Contingency analysis showed no significant differences of patient-characteristics with HRS-cells containing mutant vs. wild-type *SOCS1*. By predicted mutational consequence, mutations can be separated into those with non-truncating point mutations (‘minor’ *n* = 49/64 = 77%) and those with length alteration (‘major’; *n* = 15/64 = 23%). Subgroups did not differ in clinicopathological characteristics; however, patients with HRS-cells that contained *SOCS1* major mutations suffered from early relapse and significantly shorter overall survival (*P* = 0.03). The *SOCS1* major status retained prognostic significance in uni-(*P* = 0.016) and multivariate analyses (*P* = 0.005). Together, our data indicate that the *SOCS1* mutation type qualifies as a single-gene prognostic biomarker in cHL.

## INTRODUCTION

The majority of patients with classical Hodgkin lymphoma will achieve complete remission with current treatment strategies [1]. Stage-appropriate treatment approaches include the combination of a short course of chemotherapy and involved field radiation in early stage disease [2, 3] whereas the approach in advanced disease is a prolonged course of chemotherapy followed by positron emission tomography (PET)-guided radiotherapy [4]. The overall impressive therapy outcomes are overshadowed by subsets of patients with high-risk advanced disease (e.g. post-treatment PET positive) [4], those patients not fit for intensive treatment regimen [5] as well as elderly patients [6]. Relapsed Hodgkin lymphoma remains curable in those patients who are suitable for high-dose chemotherapy and autologous stem cell treatment [7]. Emerging new treatments, including antibody-drug conjugate brentuximab-vedotin [8] or the immunologic checkpoint inhibitors nivolumab [9] promise further substantial improvements in relapsed patients and also in first line patients in future.

All these successes are beclouded by long-term toxicities including secondary neoplasms [10], cardiac failure [11], and/or infertility [12]. In the last decade, a sizeable number of clinical trials focused on reduction of toxicity and lead to further improvement while maintaining excellent outcomes. Collectively, only post-treatment PET was established as a marker for response without additional radiotherapy in advanced Hodgkin lymphoma [4]. However, there is no reliable routine-diagnostic biomarker enabling upfront identification of cHL patients that do not show response to therapy [13, 14]. One reason may be related to the specific pathobiology of cHL with the neoplastic cells, the Hodgkin Reed-Sternberg cells (HRS), composing only a small part of the cellular tumor mass [15]. Consequently, several studies in the last decade have focused on the surrounding inflammatory infiltrate albeit with variable results—especially regarding prognostication [16]. Similarly, the high inter-rater variability and subjective nature of immunohistochemical (i.e., tissue-based HRS-cell) biomarkers precluded establishing reliable tissue-based prognostic markers [17] (NCT01505712; http://www.clinicaltrials.gov).

*Suppressor of cytokine signaling 1* (*SOCS1*) mutations have been described in specific sets of malignant lymphomas [18]. For example 35% of primary mediastinal B-cell lymphomas [19] and 16% of diffuse large B-cell lymphomas harbor *SOCS1* mutations [20]. In cHL cases, we have previously described *SOCS1* mutations in ∼45–52% [21]. The encoded SOCS1 protein inhibits janus kinase and signal transducer and activator of transcription (JAK/STAT) signaling, and the C-terminal domain including the SOCS box is necessary for this function [22, 23]. We have shown that mutations affecting this domain result in abnormal stabilization of JAK2 and dysregulation of JAK/STAT signaling [24]. While the specific pathobiological role in lymphomagenesis remains to be elucidated, *SOCS1* is a postulated tumor suppressor gene, that is frequently targeted by somatic hypermutation [18, 20, 25, 26] and inactivated by genomic mutations [21, 22, 24]. We have recently reported that the *SOCS1* mutation status in DLBCL carries prognostic significance [20]; however, despite being one of the most frequent recurrent somatic mutation in cHL, the clinical relevance of *SOCS1* mutations has not been examined.

The aim of the present study was to determine the clinical phenotype and prognostic significance of the *SOCS1* mutation status in a cohort of cHL patients. We found that *SOCS1* mutations occur in more than 60% of cHL patients and that mutational subtypes have different prognostic implications. Thus, the *SOCS1* mutation status in HRS cells represents a novel, tumor cell-derived, single gene prognostic biomarker in cHL.

## RESULTS

Our study cohort is composed of 105 histologically confirmed cases of cHL. Specifically, 100 cases were chosen as consecutively cryobanked samples. To increase statistical power, we followed a previous approach [27] and attempted to genotype 12 relapsed patients by establishing a separate 5-fragment PCR for *SOCS1* sequencing from FFPE samples (Supplementary Figure S1; Supplementary Table S1). Due to insufficient DNA-quality, we ultimately added 5 of the 12 patients with treatment failure. An overview of the study cohort is provided in Table 1 and based on the clinical characteristics we consider our cohort representative of cHL.

**Table 1:** Characteristics of patients with classical Hodgkin lymphoma in the study cohort

Characteristic	Patients (*N* = 105)	%
	No.	
**Demographics**		
Age, median (range)	28.2 (7–81)	
Female	59/105	56
Male	46/105	44
**Morphology**		
Nodular sclerosing	66/105	63
Lymphocyte rich	18/105	17
Mixed cellularity	16/105	15
Lymphocyte depleted	2/105	2
NOS	3/105	3
**Ann-Arbor Stage**		
Stage I	5/101	5
Stage II	51/101	50
Stage III	23/101	23
Stage IV	22/101	22
**Clinical Parameter**		
<3 LN-Areas	33/101	33.3
Age ≥ 45	17/105	16
B-Symptoms	47/96	49
EN-sites *N* = 0	77/101	76.2
EN-sites *N* = 1	16/101	15.8
EN-sites *N* ≥ 2	8/101	8
Spleen involved	10/101	10
Mediastinum	77/101	76
Inguinal LN	4/101	4
**Laboratory Parameters**		
EBV-positive	13/76	17
Elevated LDH >250 U/l	16/95	16.8
Leukocytosis ≥15000/μl	25/99	25
Hypoalbuminemia <4 g/dl	33/75	44
Hb. <10.5 g/dl	21/98	21
Incr. sed.-rate >50 mm/h	39/70	56
**Therapy**		
ABVD	39/101	38.6
BEACOPP	44/101	43.6
Other	18/101	17.8
Radiation (yes)	68/99	69
Radiation (no)	31/99	31

Abbreviations: EN, extranodal; EBV, Epstein-Barr Virus; ESR, erythrocyte sedimentation rate; LDH, lactate dehydrogenase >250 μg; Hb, hemoglobin; N, number of cases per genotype-specific subgroup; No., number of patients; LN, lymph node.

### Somatic *SOCS1* mutations occur in ∼60% of cHL patients

**Figure 1:**
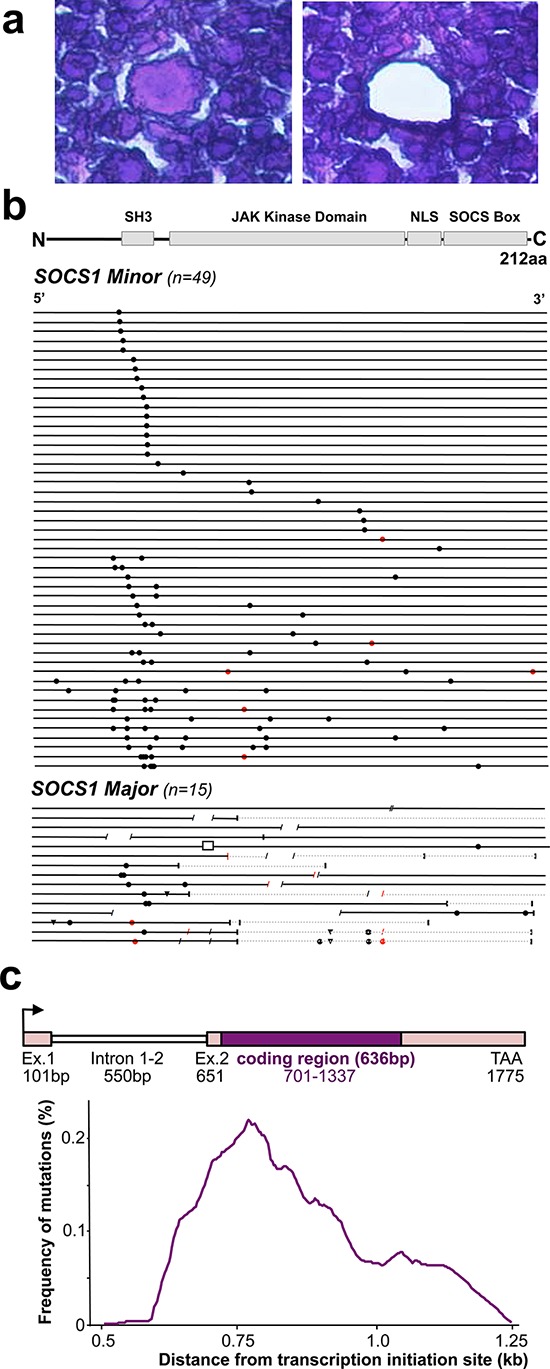
*SOCS1* Mutations in Microdissected Hodgkin/Reed-Sternberg (HRS) cells in classical Hodgkin Lymphoma (cHL) **a.** The histological composition of cHL necessitates laser capture microdissection for accurate genotyping of the neoplastic HRS-cells (center; before and after laser capture microdissection) vs. surrounding inflammatory cells. **b.** Mutational analysis of the *SOCS1* gene in laser-capture microdissected HRS cells from patients with cHL. The coding region (length: 636 bp) is shown as a black line and symbols visualize the type and site of each mutation. Mutations that do not alter the length of the encoded protein are grouped as ‘minor’ mutations whereas those HRS that harbor indels and/or truncating mutations were grouped as ‘major’. Circles are replacement substitutions, triangles are single nucleotide deletions, diagonal lines are deletions of more than one nucleotide, a box represents an insertion, and vertical lines symbolize premature stop codons followed by grey lines that represent non-sense sequence. Symbols are red when mutations occurred at sites with a consensus motif for somatic hypermutation (see methods). Abbreviations: SH3, Src homology 3; JAK, Janus kinase; NLS, nuclear localization signal; SOCS box, silencer of cytokine signaling box. **c.** Distribution of somatic mutations from the transcription initiation site. Mutation frequency indicates the number of mutation per overlapping intervals of 100 bp in the mutated cHL and is plotted aginast the distance (kb) from the transcription initiation site (arrow in scheme of genomic locus).

We laser-capture microdissected > 50–1000 HRS cells per patient sample (Figure 1a) and performed full-length sequencing of the *SOCS1* gene. We identified *SOCS1* mutations in HRS cells from 64 of 105 patients (61%). In 30 cases, we also laser-capture microdissected > 100–500 cells of the surrounding cells (‘infiltrate’); however, failed to detect *SOCS1* mutations. In conjunction, these data confirm that in cHL, *SOCS1* mutations are HRS-cell specific and the prevalence of 61% makes *SOCS1* mutations to one of the most frequent recurrent somatic mutational event in cHL. Figure 1b summarizes all mutations within the coding region of *SOCS1* (see also Supplementary Table S2).

### *SOCS1* mutations differ by length of intact coding sequence

In total, we found 140 unique mutational events in 64 separate cases (referred to as *SOCS1 mutant*). In most mutant cases (∼85.9%) the mutation was admixed with wild-type sequence (heterozygous pattern) whereas 9 of the 64 *SOCS1 mutant* cases (14.1%) showed a homozygous mutation pattern (either due to loss of the wild-type allele or a biallelic mutation). Twenty-six of the 64 *SOCS1 mutant* cases carried single point mutations (40.6%), whereas multiple mutations accumulated in 38 cases (59.4%; Figure 1b). Mapping of the *SOCS1* mutations over the coding region showed a pattern similar to that observed in other tumor suppressor genes. In comparison to the distribution of *SOCS1* mutations in other lymphomas [18, 20, 26], point mutations in cHL showed a higher prevalence in the SH3 domain, whereas deletions predominantly affected the JAK Kinase Domain (Figure 1b, Supplementary Table S2). By mutation-type we found 18 deletions, 1 insertion and 121 point mutations (single-nucleotide substitutions). The single-nucleotide substitutions were composed of 19 synonymous- and the 102 non-synonymous point mutations consisted of 99 missense and 3 non-sense mutations. We also screened for single nucleotide polymorphisms (at positions c.58, 381, 384, 421, 558, 577, 593, 595, 597, 630); however, found the wild-type allele in all microdissected HRS- and all 30 inflammatory infiltrate samples. *SOCS1* mutations rarely localize primarily to C-terminal domains; however, when consequences of upstream mutations were considered [20], the fraction of cases with predicted alterations in C-terminal domains increased substantially. The deleterious impact of truncations and/or frameshifts that alter longer stretches of the gene, affected in particular the SOCS box (range 51–100% of *SOCS1* mutant cHL). Thus, *SOCS1* C-terminal domains including the terminal part of the JAK-domain, the nuclear localization sequence (NLS) and the entire SOCS box (Figure 1b) are rarely affected by primary mutational events; however, these C-terminal domains are mutated or lost due to more severe proximal mutations [20]. Consequently, *SOCS1* sequence analysis implies different degrees of mutational severity, which can be visualized via the length of intact coding sequence (Figure 1b). Accordingly, we followed prior designations [20] and defined *SOCS1 minor* as cases that harbor only non-foreshortening point mutations (49 of 64 *SOCS1* mutated cases in our cHL cohort = 76.6%), and the *SOCS1 major* group as cases with at least one length-alternating mutational event (15 of 64 *SOCS1* mutated cases in our cHL cohort = 23.4%). To account for these differences, we performed subgroup analyses based on the two mutation subtypes (*SOCS1 minor* vs. *major)*.

### *SOCS1* mutations occur at somatic hypermutation (SHM) motifs

Prior studies have suggested that *SOCS1* mutations might be the result of SHM [18, 20, 25, 26]. When taking the untranslated exon 1 (101 bp), intron 1–2 (550 bp), and the untranslated region of exon 2 (50 bp) into account, the highest frequency of point mutations localizes to ∼0.5–0.8kb from the transcription initiation site (Figure 1C). These findings are in line with the frequency distributions found in other genes targeted by SHM [28] and may explain the relative high frequency of point mutations in the SH3 domain. Additionally, we checked the hotspot consensus motifs (RGYW/WRCY, DGYW/WRCH and WA/TW) [29–31] known as somatic hypermutation target sites that result in single nucleotide substitutions (Supplementary Table S2). We found 16 mutations occurring in these SHM motifs and notably 69% matched the RGYW motif [30], which is in line with the high prevalence of G/C substitutions (96% vs. only *n* = 5 A/T substitutions) in SHM [28]. Furthermore, in five cases at least one flanking site of a deletion matched one of the SHM consensus motifs, and in one of the major cases (total of 6 mutations), position c.429 was affected on both alleles (one by a missense mutation, the other by a deletion; Supplementary Table S2). Although comparison to mutational rates at SHM consensus motifs in cHL (*n* = 16/166 = 9%) is significantly lower than that observed in DLBCL (*n* = 33/120; 27.5%; *P* < 0.01, Fisher's exact test) [20], our data suggest that *SOCS1* mutations are at least in part caused by aberrant SHM.

### Expression of mutant *SOCS1* alleles in HRS cells in primary cHL samples

**Figure 2:**
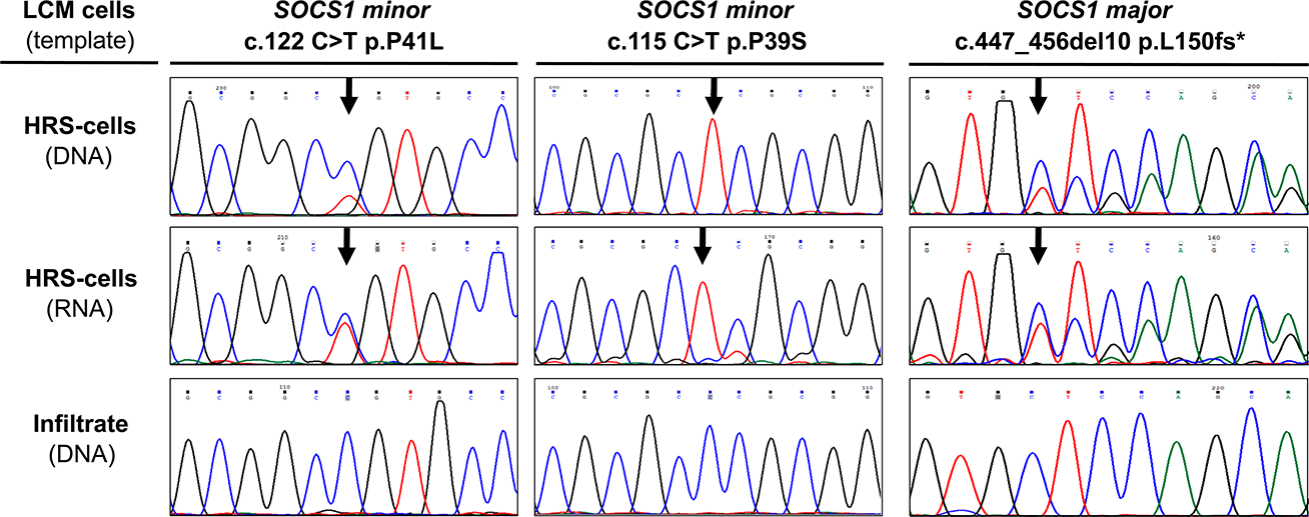
Expression of *SOCS1* Mutations in Hodgkin/Reed-Sternberg (HRS cells in classical Hodgkin Lymphoma (cHL) Sanger sequencing of nucleic acids, isolated from laser capture microdissected (LCM) cellular subsets demonstrates presence and expression of the mutant allele in HRS cells. Note: the major mutation shown here was detected in a sample genotyped at time of progression; this major mutation was not present in the primary biopsy and is therefore not included in the analysis or figure 1.

Some of the detected mutations in the coding region are predicted deleterious and likely inactivate the tumor suppressor SOCS1. However, as most HRS cells carry, in addition to a mutant, a wild-type allele (Figure 2, top traces DNA from HRS-cells) it is not clear if the mutant *SOCS1* gene is expressed. Therefore, we isolated RNA from microdissected HRS cells in 28 cases. In 14 cases yields were too low/insufficient; however, genotyping revealed expression of mutated alleles in 6 of 11 *SOCS1 minor-* and in 2 of 3 *SOCS1 major* cases. Representative examples of two *SOCS1 minor* and one *SOCS1 major* case are provided in (Figure 2). Specifically, one of the *SOCS1 minor* cases demonstrated loss of heterozygosity for the mutated base at position c.115 (Figure 2, middle) and genotyping of the corresponding RNA demonstrated expression of the mutated allele. Thus, we show that not only cHL cell lines [21], but also *SOCS1* mutated HRS cells in lymphoma tissue express the mutant allele.

### Clinical phenotype and follow-up in *SOCS1* mutated cHL

**Figure 3:**
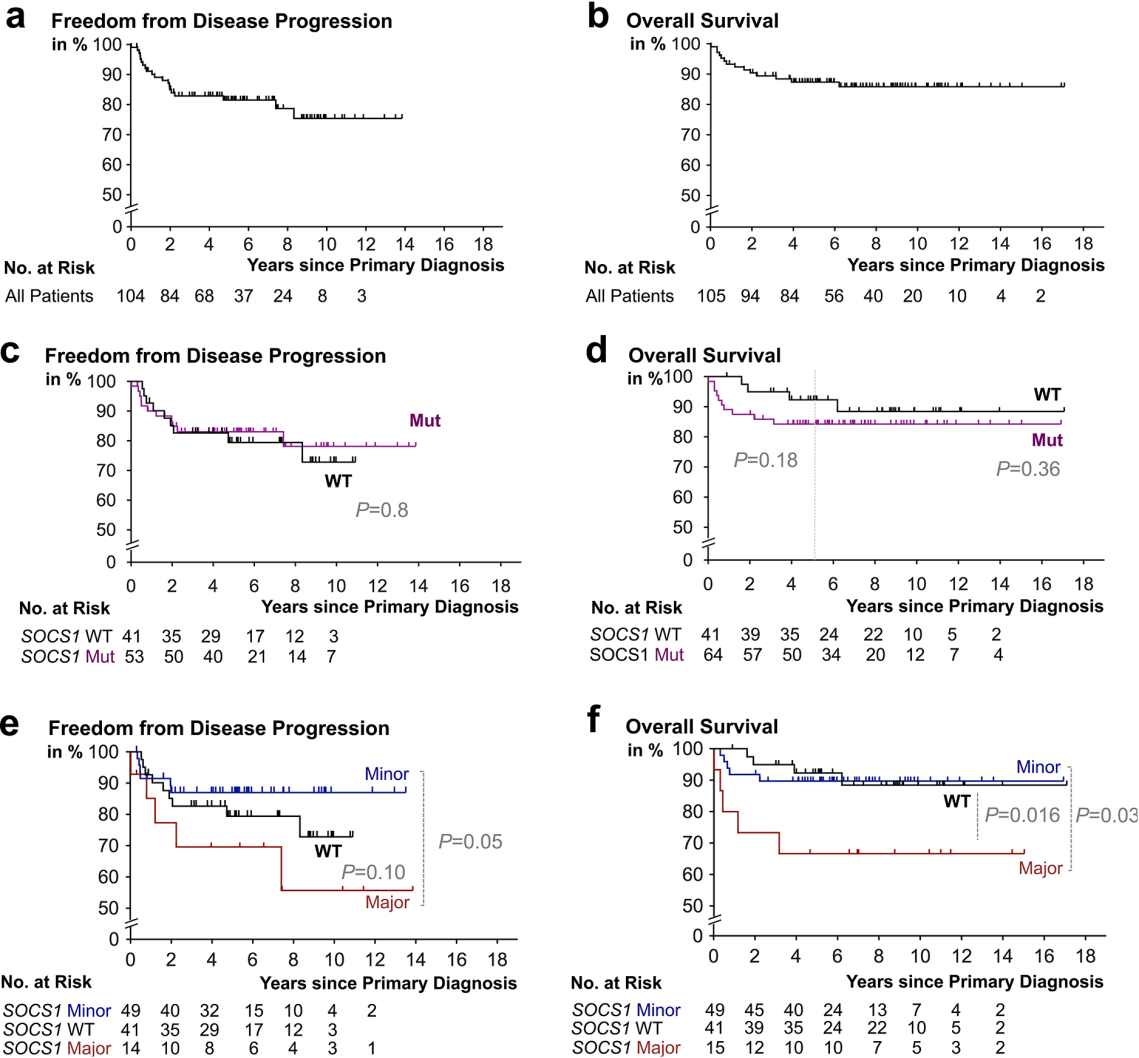
Kaplan-Meier survival estimates according to *SOCS1* mutation status Progression free **a.** and overall survival **b.** in our study cohort. **c.** Patients with classical Hodgkin lymphoma (cHL) containing HRS cells that harbor *SOCS1* mutations (Mut) mutations (purple) had a no significantly different rate of freedom from disease progression when compared to patient whose HRS-cells harbored *SOCS1* wild-type (black). **d.** cHL-patients with RS-cells that harbored *SOCS1* mutations (purple) showed earlier events; however, had no significantly shorter overall survival times when compared to patient whose HRS-cells harbored *SOCS1* wild-type (black). **e.** Patients with classical Hodgkin lymphoma (cHL) containing HRS cells that harbor *SOCS1 major* mutations (red) had a significantly different rate of freedom from disease progression (*P* = 0.05) when compared to cases with *SOCS1 minor* mutations (blue). **d.** cHL-patients with RS-cells that harbored *SOCS1 major* mutations (red) had significantly shorter overall survival times when compared to patient whose HRS-cells harbored *SOCS1* wild-type (black) or *SOCS1* minor mutations (blue; *P* = 0.03). All *P* values from log rank tests.

Contingency analysis of epidemiological, clinicopathological, and therapeutic characteristics in our cHL cohort with respect to their co-occurrence with *SOCS1* mutations, or mutation subtypes, showed no significant differences or associations (Table 2). Specifically, phenotype analysis delineated that no specific characteristic allows discrimination of either the *SOCS1* wildtype, mutant, minor, or major subgroup. Therefore, the *SOCS1* mutation status or subtype cannot be inferred from a basic panel of parameters. Importantly, the applied treatment regimens showed no significant differences when compared between mutational subtypes (Table 1, 2). At the time of data evaluation, we separated outcome by freedom from disease progression (FFDP) and overall survival (OS) (Figure 3). The median follow-up time for FFDP was 5.2 years (range: 1 month - 14 years) with 19% of patients suffering from relapse or refractory disease (*N* = 20/105; Figure 3a). The median follow-up time for OS was 6.6 years (range: 1 month - 17 years). Fourteen patients (∼13%) had died of disease and 91 of 105 patients (∼87%) were censored (either alive or lost to follow-up; Figure 3b). These outcome characteristics are comparable with previously reported outcomes [32, 33] and we, thus, consider our cohort representative for the study of prognostic biomarkers in cHL.

**Table 2:** Characteristics of *SOCS1* genotype-specific subsets of patients with classical Hodgkin Lymphoma

	*SOCS1*-wild-type *N* = 41		*SOCS1*-mutated *N* = 64		*SOCS1*-minor *N* = 49		*SOCS1*-major *N* = 15		*P*	*P*	*P*	*P*
Characteristic	No.	%	No.	%	No.	%	No.	%	WT vs. mut.	WT vs. Minor	WT vs. Major	Major vs. Minor
**Demographics**												
Age, median (range)	25.1 (7–73)		29.22 (11–81)		30.2 (11–76)		28.9 (13–81)		0.26^t^	0.25^t^	0.52^t^	0.9^t^
Female	23/41	56	36/64	56	27/49	55	9/15	60	1.0	1.0	1.0	0.76
**Morphology**									0.7^c^	0.57^c^	0.79^c^	0.52^c^
Nodular sclerosing	29/41	70.7	37/64	57.8	27/49	55.1	10/15	66.6	0.22	0.19	0.76	0.55
Lymphocyte rich	5/41	12.2	13/64	20.3	12/49	24.5	1/15	6.7	0.43	0.18	1.0	0.27
Mixed cellularity	5/41	12.2	11/64	17.2	8/49	16.3	3/15	20	0.58	0.77	0.67	0.71
Lymphocyte depleted	1/41	2.4	1/64	1.6	1/49	2	0/15	0	1.0	1.0	1.0	1.0
NOS	1/41	2.4	2/64	3.1	1/49	2	1/15	6.7	1.0	1.0	0.47	0.42
**Ann-Arbor Stage**									0.12^c^	0.11^c^	0.44^c^	0.77^c^
Stage I	1/39	2.6	4/62	6.5	4/49	8.2	0/13	0	0.65	0.38	1.0	0.58
Stage II	25/39	64	26/62	41.9	20/49	40.8	6/13	46.2	**0.04**	0.36	0.33	1.0
Stage III	5/39	12.8	18/62	29	14/49	28.6	4/13	30.8	0.08	0.12	0.2	1.0
Stage IV	8/39	20.5	14/62	22.6	11/49	22.4	3/13	23	1.0	1.0	1.0	1.0
**Clinical Parameter**												
<3 LN-Areas	10/39	25.6	23/62	37.1	16/49	32.7	7/13	53.8	0.28	0.49	0.09	0.52
Age ≥45	5/41	12.2	12/64	18.8	10/49	20.4	2/15	13.3	0.43	0.4	1.0	0.72
B-Symptoms	18/36	50	29/60	48.3	21/47	44.7	8/13	61.5	1.0	0.66	0.53	0.35
***Extranodal (EN) status***									0.7^c^	0.51^c^	0.6^c^	0.35^c^
EN-sites 0	31/39	79.5	46/62	74.2	36/49	73.5	10/13	76.9	0.64	0.62	1.0	1.0
EN-sites 1	6/39	15.4	10/62	16.1	7/49	14.3	3/13	23.1	1.0	1.0	0.67	0.42
EN-sites ≥2	2/39	5.1	6/62	9.7	6/49	12.2	0/13	0	0.48	0.29	1.0	0.33
Spleen involved	1/39	2.6	9/62	14.5	7/49	14.3	2/13	15.4	0.08	0.07	0.15	1.0
Mediastinum involved	29/39	74.4	48/62	77.4	36/49	73.5	12/13	92.3	0.81	1.0	0.25	0.26
Inguinal involved	1/39	2.6	3/62	4.8	3/49	6.1	0/13	0	1.0	0.63	1.0	1.0
**Laboratory Parameters**												
EBV-positive	4/30	13.3	9/46	19.6	8/37	21.6	1/9	11.1	0.55	0.53	1.0	0.66
Elevated LDH >250 U/l	9/37	24.3	7/58	12.1	5/45	11.1	2/13	15.4	0.16	0.15	0.7	0.65
Leukocytosis ≥15000/μl	11/38	28.9	14/61	23	11/48	22.9	3/13	23	0.64	0.62	1.0	1.0
Hypoalbuminemia <4 g/dl	11/29	37.9	12/46	26.1	6/34	17.6	6/12	50	0.31	0.09	0.51	0.05
Hb. <10.5 g/dl	5/38	13.2	16/60	26.7	12/47	25.5	4/13	30.8	0.14	0.18	0.21	0.73
ESR >50 mm/h	16/29	55.2	23/41	56.1	20/33	60.6	3/8	37.5	1.0	0.8	0.45	0.27
**Therapy Protocol**									0.7^c^	0.8^c^	0.2^c^	0.15^c^
ABVD	14/39	35.9	25/62	40.3	21/49	42.9	4/13	30.8	0.68	0.52	1.0	0.53
BEACOPP	19/39	48.7	25/62	40.3	21/49	42.9	4/13	30.8	0.42	0.67	0.34	0.54
Other	6/39	15.4	12/62	19.4	7/49	14.3	5/13	38.5	0.79	1.0	0.12	0.11
Radiation (yes)	27/38	71.1	41/61	67.2	32/49	65.3	9/12	75	0.82	0.65	1.0	0.73

Abbreviations: EN, extranodal; EBV, Epstein-Barr Virus; ESR, erythrocyte sedimentation rate; LDH, lactate dehydrogenase >250 μg; Hb, hemoglobin; N, number of cases per genotype-specific subgroup; No., number of patients; LN, lymph node; *P* values from Fisher's exact test for comparison dichotomous variables (provided per line), chi-square for comparisons when taking all categories into account (provided in the same line as category-heading and indicated by ^c^), or student *t*-test for age (indicated by ^t^).

### Outcome differs by mutational subtype

**Figure 4:**
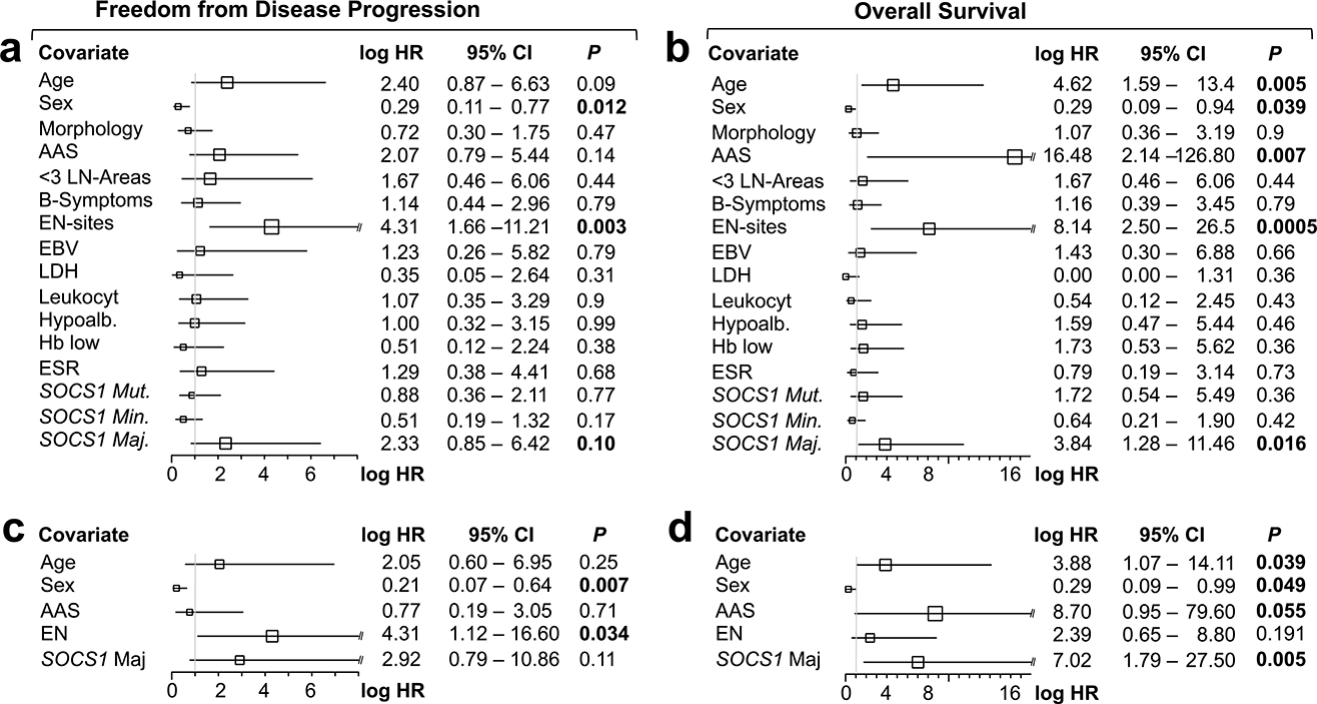
Forest plots of univariate **a, b.** and multivariate **c, d.** log hazard ratios (HR) for failure from tumor progression (a, c) and overall survival (b, d) according to epidemiological, clinico-pathological, serological covariates, and *SOCS1* gene status as well as mutation types. a, b. Univariate and c, d. multivariate Cox proportional hazards regression models. *Abbreviations*: AAS, Ann Arbor Stage; < 3 LN-Areas, number of independently involved lymph node regions; EN, extranodal; Leukocyt, leukocytosis; hypoalb, hypoalbuminemia; ESR, erythrocyte sedimentation rate. For specific cutoffs see methods or tables.

Outcome analysis comparing patients with HRS-cells harboring mutant vs. wild-type *SOCS1* showed no significant differences with respect to FFDP (*P* = 0.77; Figure 3c); however, we found a trend towards shorter overall survival in the *SOCS1* mutant subgroup (*P* = 0.36; Figure 3d); which was exaggerated when restricting statistical comparison to the initial 5 years (*P* = 0.18; Figure 3d). Comparison by mutation subtype showed that cHL patients with HRS-cells that harbor *SOCS1* minor mutations had different time courses of FFDP when compared to the *SOCS1* major subgroup (*P* = 0.05; Figure 3e); however, overall survival of *SOCS1* minor patients was similar to *SOCS1* wild-type patients (*P* = 0.88; Figure 3f). In contrast, patients with HRS-cells that harbored *SOCS1* major mutations suffered from a higher fraction of earlier relapse (Figure 3e; *P* = 0.05) and significantly shorter overall survival when compared to *SOCS1* wild-type (*P* = 0.016; Figure 3f) or *SOCS1* minor patients (*P* = 0.03; Figure 3f). We corroborated these observations in univariate comparisons of the *SOCS1* mutation status (major, minor, both) with 13 other covariates revealed that the *SOCS1* major status is associated with distinct prognostic fates. While FFDP did not reach significance (*P* = 0.10; Figure 4a), OS was significantly shorter in the *SOCS1* major subgroup (*P* = 0.016 Figure 4b). Based on these univariate comparisons, we assessed independence of the *SOCS1* major status in multivariate Cox models for FFDP and OS (Figure 4c, 4d). With respect to overall survival (Figure 4d), the *SOCS1* major status had significant prognostic information independent of the covariates age, sex, AAS, and extranodal involvement (*P* = 0.005; Figure 4d). Together these data indicate that the *SOCS1* gene mutation status had little prognostic impact by itself; however, the *SOCS1* mutation subtype, and in particular the *SOCS1* major mutations, has impact independent from the canonical prognostic biomarkers in cHL [14].

## DISCUSSION

Here, we evaluated the frequency, clinical phenotype and prognostic value of *SOCS1* mutations in HRS cells in a cohort of 105 cHL patients. We report that HRS cells harbor *SOCS1* mutations in over 60%, which is one of the most frequently mutated genes in cHL. We describe that no particular clinical phenotype is associated with *SOCS1* mutant cHL patients and found that the *SOCS1* mutation type is a single-gene prognostic biomarker in a subset of cHL patients. While confirmation in an independent cohort—ideally by a separate group—is pending, our findings have several implications:

The key hurdle in genotyping of cHL is isolation of HRS cells or more specifically their nucleic acids. We concede that laser-capture microdissection of HRS cells is time consuming (∼15 hours per case) and clearly does not qualify as routine diagnostics. Furthermore, the lack of mutational hotspots suggests that full-length sequencing of *SOCS1* is necessary to determine the *SOCS1* mutation status. The shortness of the *SOCS1* coding region (636 bp) is encouraging; however, the very high GC-content (>80%) has placed *SOCS1* already on the list of genes with poor coverage and sequencing quality—even when using high-performance next-generation genotyping [34]. Nevertheless, our study provides a manually microdissected and Sanger-sequencing based starting-point as proof of general feasibility to perform *SOSC1* genotyping in HRS cells. Methodological progress [35] will hopefully lead to detection of mutated genes in tumor tissue with low frequency of neoplastic cells. Furthermore, availability of early evidence for genotyping of cell-free DNA in patients may open up novel approaches for routine diagnostic assessment of the *SOCS1* mutation status [36].

From a biology perspective, our finding that *SOCS1* is a target of SHM in cHL has several implications. First, the key mutational features of genes affected by SHM (originally described in DLBCL [28] and subsequently verified in several lymphomas [18, 25, 26]) include: single nucleotide substitutions, occasional deletions and insertions, a preference for transitions over transversions, a preferential distribution within the RGYW motifs, and elevated ratios of G/C over A/T substitutions. Our report confirms the presence of all these features in the *SOCS1* gene in HRS cells of cHL and gives further support to the concept of aberrant SHM as a key oncogenic event in B cell neoplasia [28] although this occurs statistically less frequent than in DLBCL [18, 20].

With respect to the pathogenesis of cHL, *SOCS1* may play an interesting role in the acquisition of autonomous growth of HRS cells. Briefly, in normal B-cells, cytokine effects (e.g. IL-4) are transmitted via specific receptors (e.g. IL-4R) and their downstream signaling cascades (e.g. JAK/STAT) that collectively stimulate proliferation and clonal expansion [37, 38]. As the name implies, the normal function of SOCS1 in this cascade is to inhibit the JAK/STAT signaling pathway. We have previously shown that *SOCS1* mutations lead to sustained action of phospho-JAK2 and ultimately to constitutive activation of JAK/STAT signaling [21, 24]. Thus the acquisition of *SOCS1* mutations may render the neoplastic cell independent from extrinsic signals and thereby acquire autonomous growth in cHL. The frequency of >60% of cases suggest that *SOCS1* mutations probably are driver mutations in cHL. A recent study using flow-cytometry based RS-cell isolation in 10 cases and 2 cell lines delineated very similar *SOCS1* mutation frequencies (66.6%) [35]. Moreover, the group performed whole-exome sequencing, which allows a more comprehensive view of mutational events in cHL. The prior description of JAK2 amplification [39] and associated co-amplification of PD-L1 [40] sparked a review of the distribution of selected mutational events to each other (Supplementary Figure S2). Although at higher frequency (66.6%), *SOCS1* mutations occur in association with *JAK2*/*PD-L1* copy number gains (but not in association with other frequent mutations; e.g. *B2M*). While therapeutic implications of these associations remain to be determined [9, 41], the co-occurrence of *SOCS1* mutations with *JAK2* amplifications suggest a subset of cHL in ‘JAK2-overdrive/hyperactivation’ that may contribute to outcome differences (Figure 3).

From a clinical perspective, our key finding is that the prognosis of patients with *SOCS1* mutations depends on the nature of the mutation. We organized mutations by the lengths of intact encoded sequence, thus splitting the cases in two groups: *SOCS1 major*, which has a poor prognosis and *SOCS1 minor*, which has a prognosis similar to the *SOCS1* wild-type group. Despite being the largest *SOCS1* mutation study in laser-capture microdissected HRS-cells from cHL samples to date, and the high prevalence of mutated cases, the number of cases with events (i.e. progress, disease-related death) in the mutated subgroups is relatively small. In diseases with high cure-rates this is a common problem—especially when dealing with highly resolved molecular stratification of patients. Nonetheless, *SOCS1* as a prognostic biomarker is able to tease out the small number of patients with events (*P* = 0.045 without FFPE samples; *P* = 0.03 with FFPE samples). The estimated hazard ratios—in particular for the *SOCS1* major subgroup—are so large that the survival difference maintained statistical significance in uni- and multivariate analyses (Figure 4). Finally, we are facing the different prognostic effects of *SOCS1* major mutations in cHL (associated with shorter OS) and DLBCL (associated with longer OS) [20]. The clinical impact of *SOCS1* minor and major mutations seem to profoundly differ depending on differences in treatment regimens and/or the cellular context, i.e., the malignant B cell in DLBCL with lots of B cell functions still intact and active vs. the HRS cell in cHL with its notorious loss of B cell identity.

In summary, we report that *SOCS1* mutations occur in >60% of cHL and that the mutation subtype predict divergent outcomes in at least a subset of patients. Thus, we propose the *SOCS1* mutation status as a novel HRS cell-derived, single gene biomarker with prognostic relevance.

## MATERIALS AND METHODS

### Study population and inclusion criteria

This study includes an institutional review board-approved, retrospective archival search and analysis of a series of patients with biopsy-proven cHL seen at Ulm University Hospital/Comprehensive Cancer Center Ulm (CCCU) [42]. Inclusion criteria were: a) at least 0.5 cm^3^ fresh-frozen and cryobanked tissue where b) cHL was histological confirmed by at least two pathologists (JKL, PM) using WHO criteria [43], c) negativity for HIV, and d) treatment naïve. To increase statistical power for outcome observations we employed a previously chosen approach [27] and established *SOCS1* genotyping on FFPE material (Supplementary Table S1, Supplementary Figure S1).

### Data collection and endpoints

Medical records were reviewed to extract data on clinicopathologic features and outcomes by three of the authors (JKL, NR, AV). The primary end points in this study were overall survival (OS) and freedom from disease progression (FFDP). OS was defined as the time-span from date of diagnosis until date of death. FFDP was defined as the time-span from date of diagnosis until the date of a disease-related event, defined as progression during treatment, death during treatment (with unknown disease status), less than a complete remission after treatment and relapse after treatment, including death due to lymphoma progression after end of treatment. We censored patients at the date of last follow up when alive or lost to follow up.

### Laser-capture microdissection and extraction of nucleic acids

To procure neoplastic HRS-cells or the surrounding non-neoplastic inflammatory infiltrate (referred to as ‘infiltrate’), we performed laser-capture microdissection (LCM) using either a PALM MicroBeam IV or a PALM Robot MicroBeam system (both Carl Zeiss, Jena, Germany). We used 12 μm thick cryosections mounted on traditional glass slides covered with transparent thermoplastic film. The target cells are identified in phase-contrast, hematoxylin counterstaining, or CD30-immunohistochemistry, followed by capture into LCM-caps (adhesive cap 200 opaque; Zeiss, Jena, Germany). The laser settings for cutting were 43 mW with a focus of 85 μm for 1–2 ns at 30 pulses per second, whereas procurement was performed using 70 mW with a focus of 80 μm for 1–2 ns at 30 pulses per second. Typically, we procured  >50 up to 1000 laser-capture microdissected cells per case. For extraction of DNA and RNA we employed the PicoPure DNA extraction kit and the Pico Pure RNA isolation kit (both Applied Biosystems, Darmstadt, Germany), respectively. Initial digestion volume was 25 μl for DNA (25 μl reconstitution buffer with Proteinase K + for 3 h at 56°C) and 12 μl for RNA (2 μl for quality assessment using an Agilent 2100 Bioanalyzer and 10 μl as template for downstream RT-PCR). For RT-PCR, we employed a two-step procedure with a first RT-step using the Super Script II Reverse Transcriptase (Invitrogen, Karlsruhe, Germany) with poly-dT(15) primers (Biomers, Ulm, Germany).

### Nested PCR design and *SOCS1* sequencing

The *SOCS1* gene is composed of two exons separated by one intron (length:550 bp): exon 1 (length:101 bp) contains the 5′ UTR (untranslated region) and exon 2 (length:1124 bp) contains part of the 5′ UTR (length:50 bp), the translation initiation site (ATG position 705), the stop codon (TGA position 1340 = c.636), and the 3′ UTR. The target used for nested PCR design was the complete open reading frame (length: 636 bp). The first reaction employed previously established (external) primers [20, 21] that capture a 761 bp PCR fragment: *SOCS1*-EX2-EXT-For (5′-CAC CCC CGG ACG CTA TG-3′) and *SOCS1*-EX2-EXT-Rev (5′-CCA CAT GGT TCC AGG CAA GTA-3′). Amplification reactions were done using a Taq DNA Polymerase PCR kit (Qiagen, Hilden, Germany) with a final volume of 50 μl in a Primus96 plus thermocycler (MWG-Biotech, Ebersberg, Germany). To increase specific binding, nested PCR protocols employed a step-wise decreasing temperature program for annealing. Specifically, denaturation (at 95°C for 35s) and elongation (at 72°C for 1 min) were kept constant, whereas annealing was performed twice at 62°C (35s), twice at 60°C and 58°C followed by 35 cycles at 56°C. In the second reaction, the first PCR amplicon (2 μl) served as a template for the nested amplification of a 698 bp fragment using a pair of internal primers: *SOCS1*-EX2-IN-For (5′-GGC TGG CCC CTT CTG TAG-3′) and *SOCS1*-EX2-IN-Rev (5′-ACG GCA TCC CAG TTA ATG CT-3′). PCR products were analyzed by electrophoresis in a 2% agarose gel, stained with ethidium bromide, visualized in ultraviolet transillumination and photographed using an Alpha Imager EP (Alpha Innotech/ProteinSimple, Santa Clara, CA, USA). For each PCR reaction, we included a positive wild-type control [21] to verify amplification of *SOCS1* and a negative control containing Milli-Q water.

### *SOCS1* sequence and mutation analysis

For sequencing, the amplified products were processed by agarose gel purification using the peqGOLD Gel Extraction Kit (peqlab, Erlangen, Germany). Sanger DNA sequencing employed the *BigDye Terminator* v3.1 Kit on a 3130 Genetic Analyzer (both ABI, Carlsbad, CA). Dye signals were translated by the *KB*™ *Base Caller Software* and visualized using the *Sequencing Analysis Software* v5.4 (both ABI), ChromasPro Software (Technelysium, South Brisbane, Australia) or MacSequenceView (http://www.gotoes.org). Forward and reverse sequences were manually analyzed by blasting the obtained sequence against the human *SOCS1* sequence (ENST0000332029; *SOCS1*-001; http://www.ensembl.org). After annotation of the nucleotide alterations, sequence information was translated into protein sequence (http://www.expasy.org/translate) and alterations were mapped over the open reading frame as well as the known SOCS1 protein domains. Additionally, the DNA sequence of mutated *SOCS1* cases was used to analyze the targeting of the somatic hypermutation mechanism at specific hotspot motifs [28]. We used a DNA pattern search tool to identify somatic hypermutation hotspots (http://www.geneinfinity.org/sms/sms_DNApatterns) and for determination of the mutation distribution over the genetic locus, we employed previously established approaches [28]. Specifically, these preferred hotspots include RGYW/WRCY (G:C is the mutable position; R = purine, Y = pyrimidine, and W = A/T) [30], DGYW/WRCH (G:C is the mutable position; D = G/T/A; H = T/C/A) [31] and WA/TW (A:T is the mutable position) nucleotide pattern at both DNA strands [29]. For in silico prediction of functional consequences of amino acid substitutions we employed the two separate prediction tools SIFT (http://sift.jcvi.org/) and PolyPhen-2 (http://genetics.bwh.harvard.edu/pph2).

### Statistical analysis

Statistical analysis consisted of Fisher's exact test (association of genotype with dichotomous factors), chi-square, or student's *t*-test (comparison of means). We calculated progression-free survival from the date of first diagnosis until date of objective disease progression or death from any cause. The Kaplan-Meier method was used to estimate FFDP and OS times. We used uni- as well as multivariate Cox proportional hazards regression models to analyze survival data. Given survival times, final life status (alive or dead) and one (univariate) or more (multivariate) covariates, the regression models produce a baseline survival curve and covariate coefficient estimates with their standard errors, 95% confidence intervals, and significance levels. The covariates included in these analyses were (parenthesis provide values set to 1): age (≥45), sex (female), morphology (nodular sclerosing), AAS (III/IV), lymph node involvement (≥3 sites), extranodal involvement (≥1 site), EBV (positive), LDH (>250 U/l), leukocytosis (≥15000/μl), hypoalbuminemia (<4 g/dl), low hemoglobin (<10.5 g/dl), increased erythrocyte sedimentation rate (>50 mm/h), *SOCS1* status (mutation positive); *SOCS1* major and *SOCS1* minor. In univariate analyses, we examined covariates for their previously acknowledged prognostic impact, when applicable. In a second step, we combined factors demonstrating significance in univariate assessment in a multivariate analysis. We employed the same covariates in multivariate models to allow comparison between FFDP and OS. For composition of the heatmap (Supplementary Figure S2) that illustrates the relationship of *SOCS1* and *B2M* mutations, as well as *JAK2* and *PD-L1* amplifications, we performed an in silico analysis of a recent next-generation whole-exome sequencing study [35]. We employed the R package (http://www.r-project.org) and GraphPad Prism for data mining; in all statistical analyses we defined *P* < 0.05 as significant.

## SUPPLEMENTARY FIGURES AND TABLES




