# Supplementary material for: Suppressor of cytokine signaling 1 gene mutation status as a prognostic biomarker in classical Hodgkin lymphoma
Source: Oncotarget. 2015 Aug 20;6(30):29097–110. doi: 10.18632/oncotarget.4829 (PMC4745714; doi:10.18632/oncotarget.4829)
Supplement: Supplementary file 1 [file oncotarget-06-29097-s001.pdf]

SUPPLEMENTARY FIGURES AND TABLE

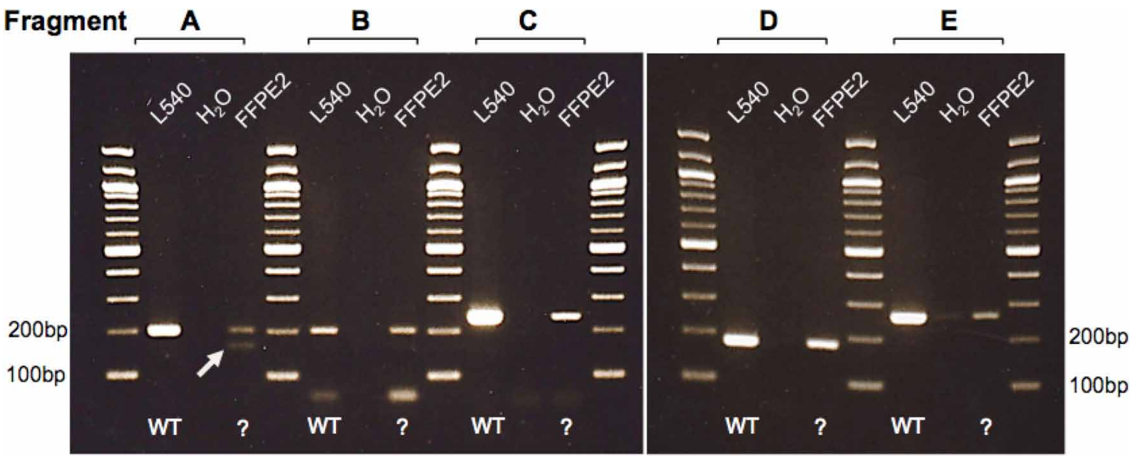

Supplementary Figure S1: *SOCS1* 5-fragment PCR-products in a control- and FFPE case. Abbreviations: bp, base pairs (ladder); WT, wild-type; ?, FFPE2 case. Order per PCR fragment (from left to right, A-E): lane 1, marker; lane 2, wild-type/positive control (WT, cellline L540); lane 3, no input DNA (= negative control; H_2O); case (FFPE2). Note the second band in fragment A (arrow1).

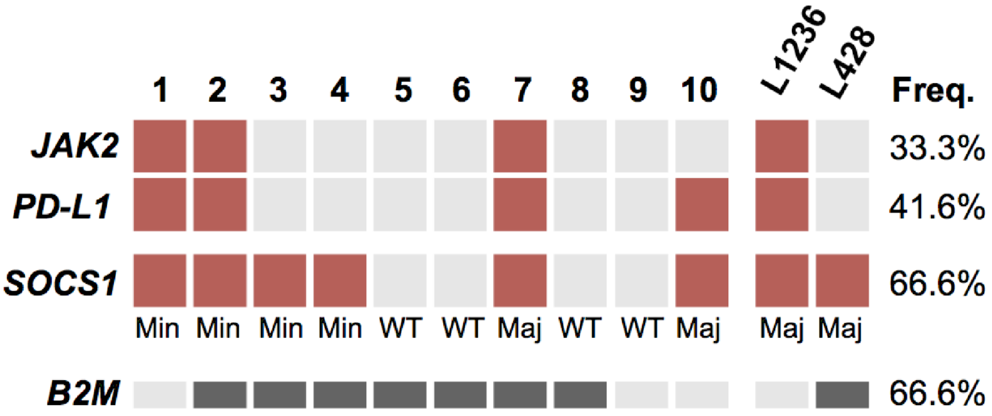

Supplementary Figure S2: Co-occurrence of *JAK2* and *PD-L1* (CD274) copy number gains with *SOCS1* mutations but not *B2M* mutations. Abbreviations: 1–10, cases; L1236, L428, cell lines; Freq., frequency; Maj., major; Min., minor; WT, wild-type.

Supplementary Table S1: Primer sequences for human *SOCS1* PCR from FFPE material

Fragment	Primer sequence	Genome position	Amplicon length (bp)
A_for<br>A_rev	5′-cac ccc cgg acg cta tg-3′<br>5′-aac gga atg tgc gga agt gc-3′	chr16:11349158-11349383	226
B_for<br>B_rev	5′-acc ttc ctc ctc ttc ctc ct-3′<br>5′-ccc cgt gca cgc tca g-3′	chr16:11349071-11349264	194
C_for<br>C_rev	5′-gca ctt ccg cac att ccg tt-3′<br>5′-tgc acg cgg atg ctc gtg gg-3′	chr16:11348950-11349177	228
D_for<br>D_rev	5′-gaa ctg ctt ttt cgc cct ta-3′<br>5′-tgg cgc agc ggg gcc ccc agc at-3′	chr16:11348833-11349009	177
E_for<br>E_rev	5′-aga gct tcg act gcc tct tc-3′<br>5′-acg gca tcc cag tta atg ct-3′	chr16:11348657-11348911	255

Abbreviations: bp, base-pairs; FFPE, formalin-fixed paraffin-embedded tissue samples; for, forward; PCR, polymerase chain reaction; rev, reverse. Genome position based on GRCh37/hg19.

Supplementary Table S2: Tables show *SOCS1* minor and *SOCS1* major cases, sorted in horizontal blocks by number of mutation per case. Columns provide for each mutation: the case number, nucleotide and predicted amino-acid change, the mutation type, SIFT (sorting intolerant from tolerant) score and prediction (http://sift.jcvi.org/), polyphen score and prediction (http://genetics.bwh.harvard.edu/pph2/), as well as somatic hypermutation (SHM) status, motif and strand.
